# Effects of Elevated Body Temperature on Selected Physiological Indices and Thermal Stress in Athletes and Non-Athletes

**DOI:** 10.2478/hukin-2021-0131

**Published:** 2022-11-08

**Authors:** Wanda Pilch, Małgorzata Żychowska, Anna Piotrowska, Olga Czerwińska-Ledwig, Wioletta Mikuľáková, Ewa Sadowska-Krępa

**Affiliations:** 1 Institute for Basics Sciences, Faculty of Physiotherapy, University of Physical Education in Krakow, Krakow, Poland.; 2 Department of Sport, Faculty of Physical Education, Kazimierz Wielki University in Bydgoszcz, Bydgoszcz, Poland.; 3 Department of Physiotherapy, Faculty of Health Care, University of Prešov, Prešov, Slovakia.; 4 Institute of Sport Sciences, Academy of Physical Education in Katowice, Katowice, Poland.

**Keywords:** active overheating, passive overheating, Physiological Stress Index, thermoregulation

## Abstract

The purpose of this study was to determine the effects of active and passive overheating. A group of athletes (A) and non-athletic men (N) underwent an exercise stress test at elevated ambient temperature and sauna bathing until an increase in rectal temperature of 1.2°C was observed. It was shown that group A performed physical exercise for a longer period of time, which elicited significantly higher amounts of work performed in the stress test. Both forms of overheating caused a significant decrease in BM and a significant change in plasma volume, while greater dehydration was observed after active overheating. Changes observed in group A were higher than in group N. MCV levels were initially higher in group A. The levels in both groups decreased after sauna bathing, although in non-athletes the decrease was greater. Both forms of overheating increased Hb, HCT, and SBP, while only the non-athletic group showed a decrease in DBP after the exercise stress test. However, a decrease in DBP was observed in both groups after sauna bathing. The PSI increased after both tests, yet to a higher extent after the stress test than after sauna bathing. The PSI was negatively correlated with VO_2max_ in both groups. The increase in cortisol concentration was higher after sauna bathing. There was a correlation between cortisol levels and the work performed during the stress test in group A. Endurance training resulted in more efficient thermoregulatory mechanisms in athletes.

## Introduction

Exercising in a hot environment, during which endogenous and exogenous heat combine, results in the accelerated onset of systemic hyperthermia. Human exercise capacity at elevated ambient temperatures is significantly restricted. The reason behind the limitation is a rapid increase in internal temperature (Ely et al., 2010) and intense sweating (Judelson et al., 2007; Stearns et al., 2009) with subsequent disruption of electrolyte (Cheuvront et al., 2003) and hormonal balance of the body (Ftaiti et al., 2008). It has been shown that the rate of internal temperature rise is very rapid when high-intensity exercise is performed in an environment with elevated ambient temperature and high humidity (Neal et al., 2016; Żuchowicz et al., 1999).

Staying in a high-temperature environment such as a sauna results in a rapid increase in skin temperature and later, despite the most efficient mechanisms of thermoregulation, heat accumulation in the body. This is evidenced by the progressive increase in internal temperature (Biro et al., 2003). High temperature affects the cardiovascular system causing accelerated circulation, blood moving from the internal vessels to the skin and an accelerated heart rate. Stress on the cardiovascular system as a result of the thermal stress response is characterized by an increase in the heart rate and cardiac output.

Evaporation of sweat from the skin surface is the main mechanism of removing excess heat from the body. During exercise, up to 80% of metabolic heat is eliminated via this route (Wilmore et al., 2008). Thermoregulatory sweat secretion occurs when the ambient temperature exceeds 28-32^o^C (Hannuksela and Ellahham, 2001). During exposure to high temperatures, about 60% of perspiration is evaporated while the remaining amount drips down without thermoregulatory significance. Skin blood flow and sweating in response to acute thermal stress can increase to as much as 7-8 l/min and 2-4 l/h, respectively, especially when the increase in body temperature results from a combination of passive overheating and physical activity (Pearson et al., 2011). Both the increase in cutaneous blood flow and the stimulation of sweat glands function show dependence on ambient temperature, skin temperature and internal body temperature (Hannuksela and Ellahham, 2001; Pearson et al., 2011).

Intense sweating causes a decrease in the body’s water reserves leading to a decrease in plasma volume and consequently blood volume (Sawka et al., 1983). This, in turn, affects the functioning of the cardiovascular system of which efficiency (transport of heat from muscles to the skin surface) affects the effectiveness of thermoregulation, in addition to such indicators as the rate of sweat secretion, hydration of the body, blood volume, ion concentration in body fluids and the level of physical activity (Sawka et al., 1983; Watt et al., 2000). Stress from overheating leads to the release of ACTH and cortisol as well as increased breakdown of endogenous proteins.

The production of heat in the body associated with physical work as well as its penetration from the high-temperature environment into the body increases physiological stress. Moran et al. (1998) developed an index allowing immediate assessment of overall physiological stress on a 0–10 scale, called the Physiological Stress Index (PSI), based on measurements of the heart rate and body temperature. Assessment of physiological stress is important for determining physiological endurance and protection against an excessive thermal stimulus of the body. The PSI reflects combined stress on the thermoregulatory and cardiovascular systems. PSI predictions are important in determining physiological endurance and protecting athletes from thermal stressors.

Kubica et al. (1996) have shown that the Polish population is characterized by high interindividual variability in the rate of the increase in body core temperature and the efficiency of thermoregulatory mechanisms, which determines the ability of the body to function in a hot environment. The interindividual variability in human exercise capacity and tolerance to high ambient temperature and humidity can therefore be measured by the time at which the lower limit of efficiency of thermoregulatory mechanisms is reached, i.e., a temperature rise of 1.2^⍛^C (Kubica et al., 1996). Therefore, it was determined that the duration of exercise that participants had to perform in an environment of elevated temperature and high humidity and the duration of sauna bathing would last until their internal temperature increased by 1.2 ^⍛^C (Tre + 1.2^⍛^C).

Żychowska et al. (2017) have found that thermal stress induces the expression of genes encoding for heat shock proteins. Differences in mRNA levels for the studied HSP-70 and HSP-27 genes between athletes and non-athletic individuals were observed. In the non-athletic male group, expression of all the assessed genes was higher than in athletes in response to the same heat stimulus. Therefore, it was concluded that the expression of genes related to heat stress depended on the level of physical activity (Żychowska et al., 2017).

In this study, it was assumed that changes in the examined indices under the influence of physical effort and the sauna would be similar within athletes and non-athletes, due to the same thermal load (an increase of internal temperature by 1.2^o^C), because the response to stress depends on the intensity of the stress stimulus, not its type (Szołtysek et al., 2011). Furthermore, adaptation to stress acquired in prolonged physical training should translate into increased tolerance of exogenous heat, hence it was hypothesized that athletes would show better tolerance of internal temperature changes, evident in lower cortisol levels and less disturbance of homeostasis. Therefore, the aim of this study was to compare the effect of physical exercise at an elevated ambient temperature of 33 ± 1°C and high humidity and passive overheating in a sauna on changes in selected hematological, physiological and biochemical indices illustrating the response of athletes and non-athletes men to exercise and thermal stress.

## Methodology

### Participants

From a group of 40 volunteers, a group of 10 non-athletic, non-smoking male students (group N) was selected. The selected men had similar levels of daily physical activity, participating twice a week for 1.5 hours in physical activities included in the study curriculum. A medical and physical examination was performed, during which resting blood pressure was measured and an ECG was recorded. Participants whose resting blood pressure was equal to or greater than 130/90 mmHg and who had an abnormal ECG were excluded from the study.

Next, maximal oxygen uptake (VO_2max_) was determined using a direct method by performing an exercise stress test on an ER 900 D- 72475 BIT 2 Jaeger cycloergometer (Friedberg, Germany). The initial warm-up load (2 min) was 60W, followed by a gradual increase of 30W every 2 min. Participants performed exercise until they were unable to maintain the imposed pedaling rhythm. During exercise, exhaled air was analyzed every 30 s (919E Medikro, Finland). VO_2max_ indices were presented as relative values (ml/kg/min). After the selection was completed, those men who had an average level of aerobic capacity according to norms were selected for the study (Arena et al., 2007).

From a group of 20 long-distance runners, 10 athletes with similar aerobic capacity (VO_2max_ 60.53 ± 13.5 ml/kg/min) and training experience (5 ± 1.2 years) were selected in a similar way as in group N. During the experiment, typical endurance training units were held an average of five times per week. During the time of recruitment into the study, athletes were in the post season and their last competition had taken place two months earlier.

Two weeks before and during the study participants did not consume stimulants such as alcohol or caffeine and did not take any vitamins or supplements.

In accordance with the requirements of the Declaration of Helsinki, study volunteers were informed about the purpose of the study, the methodology used, possible side effects, and the possibility of withdrawing from the study at any stage, without giving any reason. Participants provided written informed consent to participate in the study. The research project was approved by the Bioethics Committee for Clinical Research at the Regional Medical Chamber in Cracow – No. 202 KBL/OIL/2003.

### Study design

Prior to testing, loads at which participants would pedal in the thermoclimatic chamber were selected. Individual load selection consisted in participants performing two six-minute efforts on a cycloergometer at varying power levels between 120 and 170 W, during which VO_2_ and the heart rate were measured in the “steady state”. When selecting the load, care was taken that during the first, lower-intensity exercise, the HR was not lower than 120 beats.min^- 1^, while during the second, higher-intensity exercise, it did not exceed 170 beats.min^-1^. When the physical load exceeded the delineated HR, a correction was made, and the test was repeated on the following day. The predicted maximal exercise load (MEL) determined from two pairs of relationships (VO_2_-power and HR-power) by the extrapolation technique was further verified by the Nilsen et al.’s test (1981). The confirmation of the reliability of the results obtained (MEL) was assumed to be the duration of 105% VO_2max_ finishing effort, which should be within a narrow range from 4 to 8 minutes. All results outside the acceptable range resulted in the repetition of the test few days after the load was modified.

The program consisted of two sets of studies and involved long-distance runners (group A, n = 10) and non-athletic individuals (group N, n = 10).

The first sets of tests (active overheating, AO) consisted of participants performing an exercise stress test to assess the efficiency of exercise-induced thermoregulatory mechanisms (Kubica et al., 1996). This test consisted of pedaling on a cycloergometer with an individually determined load (53 ± 2% VO_2max_). The exercise was performed in a thermoclimatic chamber at 33 ± 1°C and 70% relative humidity until the rectal temperature was raised by 1.2°C. Participants wore shorts and athletic shoes during the exercise protocol.

The second intervention (passive overheating, PO) was performed after a one-month break. It consisted of passive heating of the body of tested participants in a Finnish sauna in which the temperature at the level of the face was 96 ± 2°C and relative humidity was 16 ± 5%. Participants stayed in the hot cabin in a semi-reclined sitting position for 15 min, followed by 2 min of cooling in a 20°C shower. These steps (15 min heating and 2 min cooling) were repeated until Tre of participants increased by 1.2°C from baseline. Participants were nude while sauna bathing.

During the first and second sets of the study, physiological indices were measured, and blood was drawn for biochemical analyses.

### Evaluation of physiological indicators

Resting SBP, DBP, and resting heart rate were measured after 30 min of rest in a sitting position on a cycloergometer in a thermoclimatic chamber before exercise, after exercise, and before and after sauna bathing. Body mass was determined before and after exercise. Participants were weighed naked with accuracy of 1g (Sartorius type F 1505 - DZA; Germany). The heart rate at five-minute intervals throughout the study was recorded using a heart rate monitor type P-3000 from Polar Elektro (Finland). Rectal temperature was monitored every 5 min using a CTD85M type electrothermometer (Ellab, Radiometer, Denmark) with accuracy of 0.1^o^C.

### Evaluation of hematological and biochemical indicators

Before and 3 min after both sets of tests, blood (15 ml) was drawn from the antecubital vein in the sitting position to BD Vauctainer tubes (New Jersey, USA). Hematological indices such as hematocrit (HCT), hemoglobin concentration (Hb) and mean corpuscular volume (MCV) were determined in blood collected on EDTA using a Sysmex XE 2100 (Kobe, Japan). Plasma samples were analyzed for total protein by the biuret method with Hitachi 917 Modular P (Tokio, Japan). Serum cortisol was measured by electrochemiluminescence with Modular E from Roche (Basel, Switzerland) (sensitivity 2μg. l^-1^, intra-assay variability CV: 2.6%, inter-assay variability CV: 6.7%).

The calculation of change in plasma volume ΔPV was made from the changes in total protein concentration determined before and after both sets of tests using the formula (Harrison et al., 1982):


$$\Delta PV=-100\cdot \left[\left(P_{f-P_{b}}\right)/\left(P_{f}\right)\right]$$

where: P_f_ - final protein determined after the test, P_i_ - initial protein determined before the test.

Cortisol concentrations measured after exercise and after the sauna were corrected after accounting for the change in plasma volume (Woźniak-Grygiel et al., 2007). The formula, according to Kraemer and Brown, was used to calculate the adjusted values (Kraemer and Brown, 1986).


$${Lc}_{c}=\left(\text{\%}\Delta {PV}^{*}0,01*Lf\right)+Lf$$

where: L_c_ – corrected level, L_f_ – level after the test

The Physiological Strain Index (PSI) was calculated from changes in rectal temperature and the heart rate according to the equation of Moran et al. (1998):


$$\begin{array}{rl} PSI= & 5\left({\;T}_{ret}-T_{re0}\right)\cdot {\left(39.5-T_{re0}\right)}^{-1}+5\left({HR}_{t}-\right.\\ & {HR}_{0})\cdot {\left(180-{HR}_{0}\right)}^{-1} \end{array}$$

where: T_re_ – rectal temperature, HR – heart rate, T_re*t*_ and HR*_t_* – simultaneous measurements taken at any time (*t*) during the exposure and T*_re0_* and HR_0_ – the initial measurements.

### Statistical analyses

Results are presented as arithmetic means ± standard deviation (X ± SD). The consistency of the distribution of the variables with the normal distribution was checked using the Shapiro-Wilk test. If the distribution did not meet the assumptions, nonparametric tests were used to test for differences in means. The Wilcoxon’s test and the Mann-Whitney U test were used to determine statistical significance between dependent and independent samples, respectively, with *p* < 0.05 established as statistically significant difference between the means. Spearman’s coefficients were calculated to assess the correlation between the indicators. All statistical analyses were performed with use of Statistica 13 software (StatSoft, USA).

## Results

The general characteristics of participants are given in Table 1. Participants in group A showed a significantly lower body fat percentage and significantly higher performance levels. Participants completed two sets of tests (exercise test and sauna bathing). The duration of the exercise test, bathing time during which rectal temperature rose by 1.2°C and the amount of work performed during exercise at elevated ambient temperature by participants of both groups are shown in Table 1.

**Table 1:** General characteristics of athletes (A) and non-athletes (N) and the average duration of the exercise test and sauna bath time.

	Athletes /A/ n = 10	Non-athletes /N/ n = 10

	$\bar{x}\pm SD$	
Age (years)	21.60 ± 0.52	21.70 ± 0.48
Body Height (cm)	179.00 ± 7.00	176.40 ± 5.44
Body Mass (kg)	67.70 ± 5.19#	73.43 ± 6.98
BMI (kg/m^2^)	21.16 ± 1.16	23.55 ± 1.92
Fat Content (%)	7.06 ± 1.54#	10.22 ± 2.23
Exercise stress test (min)	30.7 ± 8.9#	25.1 ± 9.1
Total work (kJ)	268.7 ± 13.3#	166.9 ± 51.2
Sauna bath (min)	30.6 ± 9.4	33.8 ± 9.3

# group A vs. group N (p < 0.05)

The mean sauna bathing time in which all participants achieved a 1.2°C increase in internal temperature was similar in groups A and N. The mean exercise time was significantly longer in group A and their mean work was significantly greater than in group N. Rectal temperature before the start of both sets of tests as well as after the exercise test and after the sauna in groups N and A was the same

Changes in BM after the exercise test and after the sauna bathing are shown in Table 2. There was a statistically significant loss of body mass in men in both study groups. Greater mass loss expressed as a percentage of baseline mass occurred in group A both after performing the Kubica test (AO) and after PO. Significantly lower reductions in the athletes’ body mass were indicated after PO than after the AO.

**Table 2:** Changes in body mass, hematological and physiological indicators and selected biochemical markers after performing the Kubica test (active overheating) and after bathing in the sauna (passive overheating) by athletes (A) and non-athletes (N).

	Active overheating			Passive overheating		
	(A) n = 10					
	***Before***	***After***	**Δ**	***Before***	***After***	**Δ**
BM (kg)	67.78 ± 5.35	66.98 ± 5.36*	(-)0.8 ± 0.16	68.22 ± 5.46	67.27 ± 5.44*	(-)0.95 ± 0.28#
MCV (fl)	90.95 ± 2.53#	89.99 ± 2.38*	(-)0.96 ± 0.77	89.43 ± 3.21#	88.26 ± 3.18*	(-)1.17 ± 0.27
(gHb .dl^-1^)	15.44 ± 0.78	16.33 ± 0.76*	0.89 ± 0.38	15.12 ± 0.68	16.34 ± 0.61*	1.25 ± 0.33
Hct (l.l^-1^)	46.8 ± 1.97	48.88 ± 2.43*	2.04 ± 1.55	45.70 ± 2.53	48.52 ± 2.54*	2.82 ± 0.78
(mmHg) SBP	123.0 ± 4.8	138.5 ± 10.5*	15.5 ± 10.1	114.5 ± 6.4#	117.5 ± 9.7	3.0 ± 11.8
(mmHg) DBP	77.5 ± 4.2	73.5 ± 14.5	(-)4.0 ± 16.3	70.5 ± 7.2#	64.5 ± 9.8*	(-)6.0 ± 7.5
(HR bpm)	81.4 ± 10.6	180.7 ± 9.0*	99.3 ± 10.9	69.1 ± 12.6#	122.8 ± 18.9*	53.7 ± 14.7&
Cortisol (ng.ml^-1^)	12.13 ± 2.59	15.43 ± 4.44*	3.3 ± 4.19	10.99 ± 2.02	15.45 ± 3.32*	4.46 ± 4.51&

	Active overheating			Passive overheating		
	(N) n=10					
	***Before***	***After***	**Δ**	***Before***	***After***	**Δ**
BM (kg)	72.51 ± 8.56	71.99 ± 8.46*	(-)0.52 ± 0.15	72.99 ± 9.34	72.44 ± 9.3*	(-)0.55 ± 0.40
MCV (fl)	88.27 ± 2.16	87.53 ± 2.42*	(-)0.74 ± 0.44	88.47 ± 2.94	87.26 ± 2.87*	(-)1.21 ± 0.44&
(gHb .dl^-1^)	15.26 ± 2.30	15.91 ± 0.84*	0.65 ± 0.40	15.18 ± 0.66	16.03 ± 0.71*	0.85 ± 0.52
Hct (l.l^-1^)	46.26 ± 1.97	47.95 ± 2.34*	1.62 ± 1.44	45.54 ± 2.00	47.87 ± 2.27*	2.33 ± 1.08
(mmHg) SBP	125.0 ± 7.8	140.0 ± 12.4*	15.00 ± 16.9	123.0 ± 7.8	126.0 ± 10.7	3.0 ± 10.0
(mmHg) DBP	81.5 ± 8.8	72.0 ± 13.1*	(-)9.5 ± 7.6	78.5 ± 5.8	62.0 ± 8.8*	(-)16.5 ± 5.2#
(HR bpm)	88.9 ± 8.3	167.2 ± 13.8*	78.3 ± 13.8	81.8 ± 13.6	131.5 ± 25.6*	49.7 ± 22.8&
Cortisol (ng.ml^-1^)	14.17 ± 5.91	16.61 ± 7.15	2.44 ± 4.44	13.11 ± 6.77	18.70 ± 4.03*	5.59 ± 5.89&

BM – Body Mass, MCV – Mean Corpuscular Volume, Hb – Hemoglobin, Hct – Hematocrit, SBP – Systolic Blood Pressure, DBP – Diastolic Blood Pressure, HR– Heart Rate * before vs. after (p < 0.05); # group A vs. group N (p < 0.05); & active overheating vs. passive overheating (p < 0.05)

The exercise test showed significantly greater dehydration as evidenced by a greater loss of plasma volume in athletes compared to non-athletes. Similarly, PO resulted in significantly greater plasma loss in group A compared to group N.

MCV in athletes before both sets of studies was significantly higher than in non-athletes. PO caused a significantly greater reduction in MCV in group N than in group A. After the exercise test and after the sauna, there was a significant increase in Hb and HCT in men from both study groups (Table 2).

SBP of groups A and N increased significantly after exercise at elevated temperature and after the sauna. DBP of athletes did not change after the exercise in the chamber. Significantly lower diastolic blood pressure was observed in men after the exercise test. Sauna bathing resulted in a significantly greater decrease in DPB in non-athletes compared to group A. Diastolic blood pressure after exogenous heat hyperthermia significantly decreased in both groups. The HR increased in athletes and non-athletes after performing work at elevated ambient temperature. This response was significantly greater in group A after the Kubica test than after sauna bathing. Similarly, in non-athletes, exercise produced significantly greater HR gains than sauna bathing. During exercise at elevated ambient temperature, significantly greater increases in the HR were observed in athletes who performed relatively more work than non-athletes (Table 2).

PSI values during the performance of the Kubica test were significantly higher in athletes than in non-athletes. In both groups, PSI values after sauna bathing were significantly lower than after the exercise test (Table 3). The PSI was negatively and significantly correlated (r = -0.41; *p <* 0.05) with VO_2max_ in both athletes and non-athletes.

**Table 3:** Changes in plasma volume and the physiological load index (PSI) results in athletes (A) and non-athletes (N) due to active and passive overheating

		Active overheating	Passive overheating	Active overheating	Passive overheating
		(A) n=10		(N) n=10	
**Δ % PV**	*x̅* ± SD	-10.5 ± 3.2#	-10.2 ± 3.3#	-8.0 ± 1.7	-7.0 ± 3.3
**PSI (points)**	*x̅* ± SD	7.69 ± 0.56&#	4.84 ± 0.84	6.76 ± 0.77&#	4.85 ± 1.1

# group A vs. group N (p < 0.05) &active overheating vs. passive overheating (p < 0.05)

The increase in cortisol concentration in both study groups was significantly higher after sauna bathing. Correlation of the analyzed indices showed a significant positive relationship between the increase in cortisol concentration and the work performed during the exercise test by athletes (r = 0.72; *p <* 0.05) (Table 2).

## Discussion

This study is the first one to compare the response of athletes and non-athletes to exercise in high ambient temperature and high humidity as well as Finnish sauna treatment. Despite the same increase in internal temperature (1.2°C), which was the assumption of the present study, sauna bathing time was similar in both study groups. Exercise duration in group A was longer than in participants who did not train on a daily basis; therefore, the work performed by athletes during this exercise was also significantly greater than that performed by non-athletes, which is the evidence of more efficient functioning of the thermoregulatory mechanisms of athletes.

Thus, it was confirmed that endurance training (5 years on average) of athletes improved their thermoregulatory mechanisms. This effect of training was also confirmed by other authors (Periard et al., 2015; Tikuisis et al., 2002).

Under the conditions of high temperature which prevails in the sauna, loss of heat with sweat is the most effective way to transfer heat by the body because other ways of heat exchange between the body and the environment (conduction, convection, radiation) cause heat transfer to the body, increasing its internal temperature. During routine sauna bathing, the average amount of water lost from the body with sweat is about 400-600 g (Pilch et al., 2014).

The magnitude of dehydration after both exercise and the sauna in the present study is reflected by the loss of body mass and the decrease in plasma volume observed in men from both groups, which is significantly higher in both sets of studies in group A.

Sweating efficiency depends on the ambient conditions under which the two tests were performed, namely ambient temperature and air vapor saturation (Cheuvront et al., 2003). The conditions in the thermoclimatic chamber where the exercise stress test was conducted, including high 70% humidity were not conducive to sweating.

The greater mass loss, and thus dehydration, observed in athletes may be related to their higher body water percentage and lower body fat levels. As reported by others, endurance training has a positive effect not only on better cardiovascular function by lowering the heart rate, but also on increasing the intensity of sweat secretion (Cheuvront et al., 2003), which caused more significant dehydration in the training group after both sets of tests.

A greater loss of body mass after both sets of the study was accompanied by a greater loss of plasma volume in athletes compared to individuals who did not train, which indicates better adaptation to harsh environmental conditions and exercise, as well as more efficient elimination of heat by the body of athletes.

Pokora et al. (2005) found differences in the distribution of body fluids depending on the type of the thermal load applied. According to those authors, exogenous thermal stress such as sauna bathing causes greater loss of water from the extracellular space, and only then from the intracellular space. In contrast, the endogenous load increases the contribution of cellular fluid to the total volume of water lost during exercise (Pokora et al., 2005).

In the present study, exercise and exogenous heat-induced dehydration not only led to a violation of extracellular water reserves (ΔPV) but also contributed to a decrease in intracellular water reserves, as reflected by a decrease in MCV (Table 2). A greater disruption of cellular water resources expressed by a 1.1% reduction in MCV occurred in athletes. In contrast, MCV decreased by 0.84% in non-athletes. Pre-exercise MCV was significantly higher in athletes than non-athletes, demonstrating the effect of long-term training on the ability to accumulate body water.

Under thermal stress, the acceleration of the heart rate is primarily the result of activation of the sympathetic nervous system and the action of norepinephrine released in greater amounts, as well as the result of increased internal body temperature (Luurila, 1992). A typical Finnish sauna bath causes the heart rate to accelerate to 100–160 beats.min^-1^ (Luurila, 1992). In the present experiment, a greater increase in the heart rate after sauna bathing was observed in non-athletes compared to athletes. Lower initial heart rates and smaller heart rate increments during sauna bathing in athletes were due to the activation of their cardiovascular system during chronic adaptation to training performed systematically over a 5-year period.

Physical exertion at elevated ambient temperature engages the cardiovascular system to a higher extent than passive overheating alone, which is observed by a progressive increase in the heart rate and a decrease in cardiac stroke volume (Wilmore et al., 2008). In the present study, a higher increase in the heart rate was observed in athletes than in non-athletes after exercise at elevated ambient temperature. This relationship is due to the fact that the work performed by athletes during the exercise test to achieve a 1.2°C increase in internal temperature was significantly higher than that of non-athletes and placed a greater strain on their cardiovascular system.

Overheating in the sauna in men from both groups caused a significant increase in SBP and DBP. Similarly, the increase in SBP under hyperthermia has been described by many authors (Kihara et al., 2002; Umehara et al., 2008).

In the present two sets of studies, the same 1.2°C increase in rectal temperature was set as a standard. In this case, the PSI was determined by changes in the heart rate due to exercise and sauna bathing. Higher PSI values that were positively correlated with VO_2max_ occurred in athletes who performed more work during exercise in a thermoclimatic chamber than in non-athletes under the same conditions. Similar observations were made by Pokora and Zebrowska (2016) who calculated the PSI in endurance runners, soccer players, and nonathletic individuals after standard exercise performed at a neutral temperature. The PSI in the two groups of athletes did not differ from that of non-athletes (Pokora and Zebrowska, 2016).

Other studies have shown that higher VO_2max_ values do not affect final body temperature during moderate exercise in a thermoneutral environment (Fritzsche and Coyle, 2000). However, higher VO_2max_ may influence the final body temperature rise in trained athletes during intense exercise in the heat (Mora-Rodriguez et al., 2010).

In our study, the PSI of both athletes and non-athletes was significantly higher after exercise in a thermoclimatic chamber than after the sauna. Calculation of PSI values during dry and wet sauna bathing was performed by Pilch et al. (2014). From their study, PSI values after a dry sauna in young men were similar to those in the present study, but differed from the PSI calculated after a wet sauna, where humidity was 60.5%. In the present study, exercise was performed at 30°C and 70% humidity which could also affect PSI values.

Changes in cortisol levels were observed both post-exercise, due to pre-exercise stress, factors not related to exercise and an increase in internal body temperature (Wright et al., 2012). Cortisol increases significantly after both the exercise in a hot environment (Janikowska et al., 2020; McMorris et al., 2006; Willmott et al., 2020; Wright et al., 2012) and sauna bathing (Pilch et al., 2013).

Intensity and duration of exercise are determinants of physical stress and the adrenal cortex response. It is assumed that physical exercise performed at an intensity equivalent to 70% of maximum oxygen uptake and lasting no less than 30 min, leads to an increase in blood cortisol levels, although this cannot be considered a rule (Anderson et al., 2016). Prolonged exercise and elevated ambient temperature cause a significant increase in the body’s internal temperature, increasing the risk of overheating and glucocorticoid adrenal cortical function (Cross et al., 1996; Laing et al., 2008).

In the present study, a positive correlation between the increase in cortisol concentration and the work performed during the exercise test by athletes was observed, which confirms that the greater the load, the greater the cortisol output, what indicates a stronger stimulation of the adrenal cortex in athletes compared to non-athletes (Minetto et al., 2005; Paridon et al., 2017). The increment of this hormone was significantly higher after sauna bathing than after exercise in both study groups, proving that sauna bathing is a greater stress on the endocrine system than exercise in a hot environment for both athletes and non-athletes.

Greater changes in the expression of genes related to the cellular stress response in athletes subjected to passive overheating in a sauna compared to moderate-intensity exercise were noted by Żychowska (2017).

## Conclusions

Long-term endurance training influenced athletes’ thermoregulatory mechanisms to function more efficiently, enabling them to perform more physical work at elevated ambient temperature. Adaptive changes in athletes’ bodies, acquired during prolonged physical training, did not translate into the differentiation between trained and untrained individuals in terms of exposure to the sauna. Therefore, it should be considered that the greater stress response from the endocrine system of athletes regarding passive overheating may be related to the gradual increase in heat production during physical work compared to its rapid effect from an exogenous source. This would indicate the importance of the intensity of the stimulus in the stress response of the body. The results obtained indicate the importance of not only the intensity of the stimulus, but also its intensity distribution over time in the body’s stress response. As such, sauna bathing should not be recommended for athletes on the same day as physical training, not only because of the high dehydration of the body after both exercise and the sauna, but also because of the increased stress response.

## Study limitations

The primary limitation of the study is that it shows the effects of passive and active overheating in men only. Thermoregulatory processes differ in both sexes, therefore, the results of the present study should apply only to men. The same is true for the age of our participants and body composition. The observations were conducted in a relatively age-homogeneous sample, and the body composition of participants was also relatively convergent. These two variables can also significantly affect the quality of thermoregulatory processes; hence, the presented findings should be considered as applicable to young, healthy men with normal body composition. The two treatments affecting the body were performed within a one-month interval. It was assumed that this is the time necessary for complete recovery of the studied variables to baseline and it does not constitute a study limitation for the present observations.
